# Postural Control during Progressively Increased Balance-Task Difficulty in Athletes with Unilateral Transfemoral Amputation: Effect of Ocular Mobility and Visuomotor Processing

**DOI:** 10.3390/ijerph17176242

**Published:** 2020-08-27

**Authors:** Michał Zwierko, Piotr Lesiakowski, Teresa Zwierko

**Affiliations:** 1 Department of Health Sciences, Wroclaw Medical University, 50-367 Wroclaw, Poland; michal.zwierko@gmail.com; 2 Department of Physical Education and Sport, Pomeranian Medical University, 70-123 Szczecin, Poland; lesiakowskipiotr@gmail.com; 3 Institute of Physical Culture Sciences, Laboratory of Kinesiology in Functional and Structural Human Research Centre, University of Szczecin, 70-240 Szczecin, Poland

**Keywords:** amputee soccer players, balance, saccades, Vienna Test System

## Abstract

This study examined postural control during single leg stance test with progressively increased balance-task difficulty in soccer players with unilateral transfemoral amputation (n = 11) compared to able-bodied soccer players (n = 11). The overall stability index (OSI), the anterior/posterior stability index, and the medial/lateral stability index during three balance tasks with increasing surface instability were estimated. The oculomotor and visuomotor contribution to postural control in disabled athletes was analyzed. Oculomotor function, simple and choice reaction times, and peripheral perception were assessed in a series of visuomotor tests. The variation in OSI demonstrated significantly greater increases during postural tests with increased balance-task difficulty in the able-bodied soccer players compared to amputees (F_(2,40)_ = 3.336, *p* < 0.05). Ocular mobility index correlated (*p* < 0.05) with OSI in conditions of increasing balance-task difficulty. Moreover, speed of eye-foot reaction has positive influence (*p* < 0.05) on stability indexes in tasks with an unstable surface. Amputee soccer players displayed comparable postural stability to able-bodied soccer players. Disabled athletes had better adaptability in restoring a state of balance in conditions of increased balance-task difficulty than the controls. The speed of visuomotor processing, characterized mainly by speed of eye-foot reaction, significantly contributed to these results.

## 1. Introduction

Postural control is one of the most highly affected functional abilities in lower limb amputees [1,2,3,4]. Usually, postural control has been characterized as the act of maintaining, achieving or restoring a state of balance during any posture or activity [5]. Deficits of balance limit the mobility of individuals with lower limb loss, resulting in a decrease in their physical capacities [6,7], daily activities [8] and, in consequence, their quality of life [9]. The loss in the lower extremity requires activation of adaptation mechanisms based on new movement patterns and adjustment strategies to regain balance control during standing and locomotion [2,10,11]. Study results of adjustment strategies for balance control have demonstrated the important role of integration among visual, vestibular, and proprioception. Bolger et al. [3] indicated that individuals with lower limb amputation used asymmetric interlimb force coordination strategies to retain balance. Moreover, it has been documented that lower limb amputees have a greater postural instability when visual cues are occluded [12,13,14]. Jiang, et al. [15] stated that individuals with lower limb amputation exhibited reduced cortical thickness in the visual cortical area V5/MT+ (involved with motion perception and perception in peripheral space) and these structural changes were negatively correlated to the time since amputation. 

The requirements related to adjustment in visual and movement strategies in amputees increase during their participation in sporting activities, particularly in sports with open-skills demand. Amputee soccer is a type of disabled sport designed for individuals with lower limb amputations in relation to the outfield players who use bilateral forearm crutches for locomotion. Players demonstrate superior motor performance, specifically related to core stability, balance and muscular strength [16,17,18]. During fast offensive and defensive actions in the game, players adapt their neuromuscular responses to static and dynamic conditions of movement and, simultaneously, fixation location of the ball, opponents and partners. High movement demands, coupled with multiple visual stimuli and moving objects in the field of view, make amputee football a discipline with high visuomotor competences. From a practical perspective, it is important to understand the visuomotor processing performed by amputees participating in such sporting activities.

Although there is evidence of the importance of adjustment strategies for postural control, including biomechanical factors, movement and sensory strategies, fixation location, motor control, and cognitive processing in able-bodied individuals [19], the contribution of these mechanisms to athletes with lower limb amputation is not yet well studied. Therefore, the aim of the present study was to analyze: (1) postural stability during single leg stance test with progressively increased balance-task difficulty in amputee soccer players compared to able-bodied soccer players; and (2) the relationship between visuomotor processing efficiency and oculomotor function to static and dynamic balance performance in amputee soccer players. In accordance with previous reports [1,10], it was expected that postural control in amputee soccer players may be impaired in comparison to able-bodied controls. In line with previous findings suggesting that eye movement strategies are important in balance control [20,21,22], and that visuomotor reaction time makes a significant contribution to postural stability [23,24], we hypothesized that high efficiency of oculomotor and visuomotor reaction will be associated with better balance control in amputee soccer players.

## 2. Materials and Methods 

Eleven athletes with unilateral lower limb amputation were recruited to this study (average age: 27.45 ± 5.2 years (range 20–33), average height: 171.81 ± 5.36 cm, average weight: 77.9 ± 8.69 kg). Athletes were members of the national amputee soccer team. The average sport experience after amputation was 8.27 ± 3.63 years (range 4–15). The major causes of lower limb amputation were traffic accidents (n = 8), then vascular disease (n = 2) and cancer (n = 1). The control group comprised 11 able-bodied soccer players participating in regular games throughout the season in the semi-professional level IV league (average age: 21.91 ± 3.11 years (range 18–28), average height: 179.81 ± 9.61 cm, average weight: 77.9 ± 8.69 kg) with an average sport experience of 11.36 ± 2.77 years (range 8–15). An a priori calculation for a repeated measures ANOVA with between and within factors using the G*Power 3.1 software (Heinrich-Heine-Universität Düsseldorf, Düsseldorf Germany) [25] was performed to calculate the minimum required sample size. This analysis was based on an assumed effect size of 0.3, alpha of 0.05, power of 0.80, two experimental groups, three measurements, and a level of correlation between measures of 0.5 [26], which projected a necessary total sample size of 20 participants. In this study, twenty-two participants composed the study sample 

The study was conducted in accordance with the Declaration of Helsinki and approved by the local bioethical committee (No. 138/17). Before examination, subjects were informed about the testing protocol. All subjects signed a written informed consent and were permitted to withdraw from the study at any time. 

### 2.1. Postural Control Evaluation

The Biodex Balance System SD (Biodex Medical Systems Inc., Shirley, NY, USA) was used to evaluate static postural control in conditions of visual feedback (eyes open). This system has 12 dynamic stability levels, with level 12 being the most stable (rigid) and level 1 the most unstable. Levels 12 to 1 provided a full 20 degrees of surface tilt. Static postural control was assessed during single leg stance on rigid platform (level 1). Next, postural control was measured during two tasks: (i) single leg stance with decreasing platform stability - levels 8 to 4; and (ii) single leg stance with platform stability at level 4. Able-bodied soccer players used the preferred leg to perform the postural control tasks.

Test duration for each of three balance tasks was 80 s (three trials of 20 s each, with a rest interval of 10 s between each). For all trials, participants were tested barefoot. During testing, participants looked straight ahead with their arms folded along their chest. Before testing, three trials of 20 s of adaptation in 12, 8, and 4 level of platform stability were performed. The overall stability index (OSI) (°), the anterior/posterior stability index (API) (°), and the medial/lateral stability index (MLI) (°) were analyzed. Higher scores of stability index indicate poorer balance control. 

### 2.2. Oculomotor Mobility Evaluation

The study involved a free-viewing visual search task without a sport-specific design, in which participants were required to detect a target (red letter E) among 47 distractors (inverted red letter E “Ǝ”, blue letter E, and red letter F), according to the procedure described by Zwierko et al. (2018). Sixteen visual search trials with randomly placed letters were conducted, of which eight contained the target. Participants stood at 1 m distance from the screen (amputee soccer players stood with crutches) and were positioned such that the center of the screen was aligned vertically with the center of the eyes, and horizontally with the nose. During the visual search task, participants used one button to confirm the detection of the target (target present trials – press button with left thumb) and another button to note the absence of the target (target absent trials – press button with right thumb). Eye movements during the visual search task were recorded binocularly using a mobile eye tracking system SMI ETG 2w operating at 60 Hz (SensoMotoric Instruments, Teltow, Germany). A standard three-point SMI calibration was carried out binocularly. Data were encoded through the iViewETG version 2.2 software (SensoMotoric Instruments, Teltow, Germany). Gaze data were analyzed using SMI BeGaze 3.5.101 software software (SensoMotoric Instruments, Teltow, Germany). Saccades were measured using the saccade detection algorithm supplied by SMI Research. Fixation was defined as a stable eye position maintained for at least 80 ms. The ocular mobility index (%) (100 x (saccade duration/(fixation duration+saccade duration)) according to Poiroux, et al. [27] was calculated.

### 2.3. Visuomotor Processing Evaluation

For visuomotor processing evaluation, the battery of the Vienna Test System (Dr Schuhfried Medizintechnik GmbH, Vienna, Austria) was used. The study evaluated simple eye-hand reaction time, choice eye-hand reaction time, and peripheral perception test.

The reaction time test for simple visual stimuli was used to assess the response rate. In this task, 28 light stimuli (yellow light) with randomly generated stimulus in the time interval of 2.5–6.0 s were used. The stimulus emission time was one second. The participants were instructed to perform a key-press in response to the programmed visual stimuli. The program registers the time of reactions, from which the following scores were calculated: reaction time - the period of time between the appearance of the stimulus and the start of movement releasing the “waiting key” (ms); motor time-the period of time between releasing the “waiting key” and pressing the “reaction key” (ms); and speed of eye-hand reaction - the period of time between the stimulus and pressing the “reaction key” (ms).

To evaluate the complex response, the test of measuring response time to chosen stimuli (form S4) was used. The task comprised 48 different stimuli in a random combination of yellow, red light, and a sound signal. Of all stimuli, only 16 required a response. The participants reacted by pressing the “reaction key” as soon as possible only when the red and yellow lights appeared on the screen simultaneously. The stimulus duration was 1.2 s. and the waiting period was in the range 1.5–4.0 s. The reaction time (ms), motor time (ms), and speed of eye-hand reaction (ms) were determined.

The peripheral perception test consisted of two subtasks performed simultaneously: a central tracking task and a peripheral perception task. Tracking was controlled by steering a “view-finder” with knobs, in such a way that the view-finder was linked to a red point on-screen. The proper position of the view-finder was confirmed by the flicker of the point. In the test, the system automatically controlled the correct positioning of the head and eyes in relation to the monitor. The test included two tasks. The first was the central tracking task, which focuses the attention of the subject in the center of the field of vision; this is based on controlling the cursor (green ring) with a special joystick located on the control panel. The aim was to maintain the cursor position so that the red balls moving horizontally on the screen were inside the view-finder; improper tracking of the ball was manifested by the flickering of an object on the screen. At the same time, the participant (sitting in front of the test apparatus) was tasked with responding in a timely manner to visual stimuli in the form of green glowing vertical diode lines appearing in the lateral field of view on special horizontal LED screens. To react to the appearance of a visual stimulus, the participant pressed a foot pedal below the apparatus as quickly as possible. The whole test consisted of 80 evoked stimuli, of which 40 occurred on the left, and 40 on the right side of the field of vision. Three parameters were included in the analysis of the results: visual field (°), speed of eye-foot reaction (ms), and tracking deviation (pixels).

### 2.4. Statistical Analysis

The Kolmogorov-Smirnov test was used to assess the normality of distribution of all investigated parameters; the data were found to be normally distributed (*p* > 0.2 for all cases). Levene’s test was also applied, demonstrating that the variances were not significantly different between groups (*p* > 0.05 for all cases). Characteristics of parameters were described as mean and SD. The dependent measures (OSI, API and MLI) were submitted to a repeated-measures mixed ANOVA with one between-subjects factor: Group (amputee soccer players, able-bodied soccer players) and one within-subjects factor: Test (1. static postural control; 2. postural control at stability level of 8 to 4; 3. postural control at stability level of 4, i.e., OSI_1 vs. OSI_2 vs. OSI_3, API_1 vs. API_2vs. API_3, for, MLI_1 vs. MLI_2 vs. MLI_3). Partial eta-squared (ηp^2^) values were reported to determine effect size. Post-hoc tests were performed using a Bonferroni correction, with a *p*-value < 0.05 considered significant. The magnitude of effect sizes for pairwise comparisons were also determined using Cohen’s d calculated as difference between the means divided by their standard deviation. Effect size (Cohen’s d) was characterized as small (0.2), medium (0.5), or large (0.8) [28]. The relationship between balance control and oculomotor function and visuomotor parameters in amputee soccer players were assessed by Pearson (*R*) correlation. Statistical significance was set at *p* < 0.05.

## 3. Results

Three different conditions for evaluation of postural control were used, i.e., 1. postural control during single leg stance on rigid platform (OSI_1, API_1, MLI_1); 2. postural control during single leg stance with decreasing platform stability at levels 8 to 4 (OSI_2, API_2, MLI_2); 3. postural control during single leg stance with platform stability at level 4 (OSI_3, API_3, MLI_3). 

### 3.1. Effects on OSI

**Figure 1:**
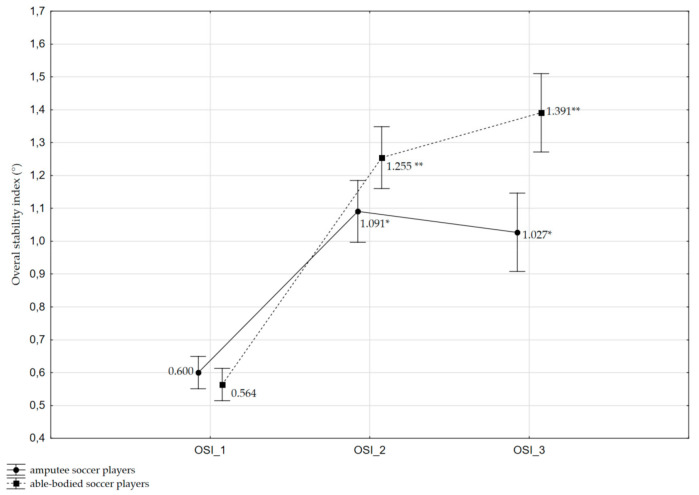
Interaction between Group and Test repetitions for overall stability index (OSI) (F_(2,40)_ = 3.336, *p* < 0.05, ηp^2^ = 0.143). Subsequent values of OSI in amputee soccer players and able-bodied soccer players are presented as means ± standard error. Significant intragroup differences between postural control tests (OSI_1 vs. OSI_2 and OSI_1 vs. OSI_3) are denoted with * (*p* < 0.01) and ** (*p* < 0.001).

There was a significant interaction between the Test and the Group factors with regard to the OSI parameter [F_(2,40)_ = 3.336, *p* = 0.05, ηp^2^ = 0.143]. It was observed that the OSI value demonstrated significantly greater increase during postural tests with increased balance-task difficulty in the able-bodied soccer players than in amputee soccer players. Specifically, compared to the static condition, there was a significant increase in OSI mean values: (1) in the postural test with decreasing platform stability at levels 8 to 4, respectively for amputee soccer players by +0.491° (OSI_2 = 1.091 ± 0.362 vs. OSI_1 0.600 ± 0.190, *p* = 0.001, effect size = 1.277) and for controls +0.691° (OSI_2=1.255 ± 0.254 vs. OSI_1 = 0.564 ± 0.129, *p* < 0.001, effect size = 2.291); and (2) a significant increase in OSI mean values in the postural test with decreasing platform stability at level 4 for disabled athletes by +0.427° (OSI_3 = 1.027 ± 0.307 vs. OSI_1 = 0.600 ± 0.190, *p* = 0.005, effect size = 1.542) and for able-bodied players by +0.827° (OSI_3 = 1.391 ± 0.468 vs. OSI_1 = 0.564 ± 0.129, *p* < 0.001, effect size = 1.526). However, there were no significant differences (*p* > 0.05) between OSI_3 and OSI_2 values in both groups of athletes, with the types of variance differing in the compared groups. The plot of interactions for the analyzed factors with regard to the OSI is illustrated in Figure 1.

### 3.2. Effects on API

There was a main effect of Test factor on API [F_(2,40)_ = 39.544, *p* < 0.001, ηp^2^ = 0.664]; API mean value increased in subsequent postural control tests in both groups. In amputee soccer players the mean difference between API_2 and API_1 was +0.336° (API_2 = 0.745 ± 0.308 vs. API_1 = 0.409 ± 0.145, *p* = 0.002, effect size = 1.114) whilst API_3 and API_1 was +0.346° (API_3 = 0.755 ± 0.225 vs. API_1 = 0.409 ± 0.145, *p* = 0.001, effect size = 1.632); in able-bodied soccer players, the index increased +0.445° (API_2 = 0.836 ± 0.250 vs. API_1 = 0.391 ± 0.104, *p* < 0.001, effect size = 1.479) and +0.582° (API_3 = 0.973 ± 0.304 vs. API_1 = 0.391 ± 0.104, *p* < 0.001, effect size = 1.789), respectively. However, there were no significant differences (*p* > 0.05) between API_3 and API_2 values in both groups of athletes. Data showed that API was not significantly different between groups in any subsequent postural control tests [F_(1, 20)_ = 1.603, *p* = 0.220, ηp^2^ = 0.074]. The plot of interactions for Group and Test in API was not statistically significant [F_(2,40)_ = 2.225, *p* = 0.121, ηp^2^ = 0.100]. 

### 3.3. Effects on MLI

Similarly to the API variation, there was a main effect of Test factor on MLI [F_(2,40)_ = 33.155, *p* < 0.001, ηp^2^ = 0.624]; post-hoc tests showed that MLI mean value increased in dynamic test conditions in comparison to static test in both groups. Specifically, in amputee soccer players the mean value of MLI_2 was 0.273° higher than MLI_1 (MLI_2 = 0.627 ± 0.241 vs. MLI_1 = 0.354 ± 0.113, *p* = 0.018, effect size = 1.003) and MLI_3 was 0.310° higher than MLI_1 (MLI_3 = 0.664 ± 0.206 vs. MLI_1 = 0.354 ± 0.113, *p* = 0.005, effect size = 1.663). Able-bodied soccer players in relation to the MLI_1 evaluation demonstrated an increase of 0.418° in MLI_2 measurement (MLI_2 = 0.736 ± 0.143 vs. MLI_1 = 0.318 ± 0.075, *p* = 0.006, effect size = 2.288) and an increase of 0.537° in MLI_3 measurement (MLI_3 = 0.855 ± 0.288 vs. MLI_1 = 0.318 ± 0.075, *p* = 0.001, effect size = 1.631). There were no differences (*p* > 0.05) between MLI_3 and MLI_2 values in both compared groups. The MLI was not significantly different between Groups (F_(1, 20)_ = 2.900, *p* = 0.104, ηp^2^ = 0.127). Consequently, there was no significant effect of interaction between analyzed factors (F_(2,40)_ = 2.168, *p* = 0.128, ηp^2^ = 0.098).

Significant correlations were found between oculomotor function parameters and postural control (Table 1). In postural control during increased balance-task difficulty, increased ocular mobility index was associated with decreased (improving) OSI_2 (R= −0. 656; *p* < 0.05) as well as the API_2 (R = −0. 624; *p* < 0.05) and API_3 (R = −0. 648; *p* < 0.05).

Positive correlations were found between visuomotor scores and overall stability index for the postural control task with increasing instability (platform stability levels 8 to 4), i.e., MLI_2 was associated with motor time (R = 0.607; *p* < 0.05). Moreover, speed of eye-foot reaction in the peripheral perception test has a positive influence on four of six parameters in postural control tasks during increased balance-task difficulty, specifically on OSI_2 (R = 0.683; *p* < 0.05), API_2 (R = 0.691; *p* < 0.05), OSI_3 (R = 0.621; *p* < 0.05), and on MLI_3 (R = 0.634; *p* < 0.05). This suggests that a lower value of postural stability indexes was associated with shorter visuomotor processing times in reaction tests, characterized by the speed of eye-foot reactions. 

## 4. Discussion

The present study investigated the effects of transfemoral lower limb amputation on postural control in athletes. Three different balance conditions were tested. Moreover, we analyzed the contribution of selected oculomotor as well as visuomotor parameters to postural control in disabled athletes. The first hypothesis, that postural control in amputee soccer players may be impaired in comparison to able-bodied controls, was not confirmed. There were no significant differences between groups in postural control parameters in relation to stability indexes. Moreover, a different plot of variation was observed in compared groups of athletes; specifically, amputee soccer players showed greater adaptability in postural tasks during increased balance-task difficulty than the able-bodied soccer players. 

On the one hand, previous studies have shown that participation in sports and/or regular physical activity has a positive effect on many aspects of amputee physical health, including cardiopulmonary, muscle strength, anaerobic power, balance and body composition [18,29,30,31], as well as social and psychological aspects of well-being [32,33]. On the other hand, it has been reported that sitting volleyball players with unilateral transfemoral amputation demonstrated poorer postural control in both static and dynamic tasks when compared to physically active non-amputees [1]. It seems that the main explanation of our study results may be the specificity of the sport activity. As opposed to seated volleyball training, where movement primarily occurs in the trunk and proximal region of the lower limbs, amputee soccer players use bilateral forearm crutches for locomotion (without prosthesis) and kick the ball only with their sound leg. 

Amputee soccer training has a positive effect on health and lowered risk of risk sports-related injuries [34]; this sport requires superior motor performance, characterized by high levels of anaerobic capacity, muscular strength, sprint performance, balance, and locomotor skills in a dynamically changing, unpredictable, and fast-paced environment [16,17,35,36]. Moreover, in line with our findings, Yazicioglu et al. [18] observed better postural control in static balance test in amputee soccer players compared to the sedentary group; however, their study group was composed of athletes with unilateral below-knee amputation. It seems that the practice of soccer may have a positive effect on proper postural maintenance in lower limb amputees as a possible result of motor cortex reorganization in amputees [37] and altered movement strategy to sustain stability balance tasks due to reduced proprioception on the amputated limb [4]. In unilateral lower limb amputees sensory changes have been observed in the non-amputated limb, suggesting that central sensory adaptations occur after amputation [38]. 

The second hypothesis of this study was that visuomotor reaction time significantly contributes to postural stability, and that high efficiency of oculomotor and visuomotor reaction will be associated with better balance control in amputee soccer players; eight significant correlation coefficients confirmed our hypothesis. First, oculomotor function parameters were associated with postural control, in particular during tests with increased balance-task difficulty; increased ocular mobility index was associated with improving the overall stability index (OSI_2), as well as the medial-lateral indexes (API_2 and API_3). Movement strategies are highly dependent on sampled visual information during balance tasks [12,13], and there is strong evidence to suggest that oculomotor control is important in this process [20,21,22]. Our study results suggest that saccade acceleration, as a rate of change of eye velocity, and saccade velocity, have no impact on postural stability during quiet standing in amputees. On the contrary, it has been reported that, overall, saccades decrease body sway in healthy individuals during simultaneous measurement of static postural stability and eye movements [21,39]. 

Moreover, our findings showed that the efficiency of ocular mobility, as a parameter describing gaze pattern, significantly contributes to adaptation in restoring a state of balance to disturbances that occur when increasing platform instability in balance tasks. It this context, exercise programs could be recommended for improving postural control during a variety of visual search tasks. However, we are aware that due to procedural limitations, our data cannot directly explain the relationship between oculomotor control and postural stability scores in amputees; this awaits further study.

The significant relationship between balance parameters in amputee soccer players and their speed of eye-foot reaction to peripheral stimulus suggests that one-leg standing is regulated by a feedback-based mechanism in which visual sensory information becomes important for continuous movement adjustments [23]. Moreover, our findings suggest that visual motor speed, which is a measure of visuomotor processing, seems to be an important element in balance control, particularly during postural disturbance. Balance requires quick adjustments to minor postural disturbances during locomotion with crutches, especially during soccer games when higher single-leg running speed is associated with an increased forward tilt of the pelvis and a shorter crutch stance phase [40]. Our study results are in line with previous observations that cognitive factors and fast processing speed were positive predictors of balance in both neurorehabilitation and the elderly [41,42]. Furthermore, there is evidence that athletes in open-skills sports exhibit better performance in visual sensorimotor processing as a result of modulation through extensive physical training [43,44,45]. Therefore, it seems that the practice of soccer may result in improved visuomotor skills in lower limb amputee subjects.

It is important to note certain aspects that may limit the present findings. First, because of the relatively small number of participants in each group, and the cross-sectional design of our study, establishment of a clear causal relationship between the long-term effects of soccer training and postural control in amputees is precluded. Second, all tests were performed in the laboratory setting, thus, it is difficult to determine the ‘‘real life’’ validity of our findings. Moreover, using alternative tools for simultaneous assessment of postural control and visuomotor efficiency in specific soccer game conditions may increase the validity of the results.

## 5. Conclusions

Our findings demonstrate that amputee soccer players display comparable postural stability to able-bodied soccer players. Moreover, amputee soccer players had better adaptability in restoring a state of balance in conditions of increased balance-task difficulty than the able-bodied soccer players. Speed of visuomotor processing, characterized mainly by speed of eye-foot reaction, have significantly contributed to these results. From a practical perspective, these results offer a chance to understand the contribution of visuomotor processing to postural stability with regard to the specific requirements of disabled sports and highlight the importance of a laboratory assessment in a sports context. We hypothesize that adaptive changes to postural control in unilateral transfemoral lower limb amputation athletes may have a connection with enhanced demand for visual motion perception and coordination during extensive practice and participation in soccer training.

## Figures and Tables

**Table 1:** Pearson correlation coefficient for postural stability, oculomotor function, simple reaction time, choice reaction time, and peripheral perception scores.

Parameters	Static Postural Control			Postural Control during Decreasing Platform Stability at Levels 8 to 4			Postural Control during Platform Stability at Level 4		
	OSI_1 (°)	API_1 (°)	MLI_1 (°)	OSI_2 (°)	API_2 (°)	MLI_2 (°)	OSI_3 (°)	API_3 (°)	MLI_3 (°)
**Oculomotor Function**									
Saccade acceleration (°/s^2^)	0.281	0.310	0.224	−0.059	−0.083	−0.011	−0.031	0.076	−0.132
Saccade velocity (°/s)	0.303	0.337	0.261	−0.046	−0.098	−0.077	−0.007	−0.053	−0.125
OMI (%)	0.064	−0.028	0.107	−0.656 *	−0.624 *	−0.549	−0.587	−0.648 *	−0.427
**Simple Reaction Time Test**									
Reaction time (ms)	0.234	0.216	0.224	0.384	0.271	0.416	0.193	0.230	0.124
Motor time (ms)	0.230	0.260	0.231	0.427	0.243	0.607*	0.310	0.206	0.344
Speed of eye-hand reaction (ms)	0.363	0.268	0.258	0.457	0.295	0.571	0.280	0.248	0.256
**Choice Reaction Time Test**									
Reaction time (ms)	0.190	0.145	0.195	0.183	0.104	0.265	0.154	0.039	0.220
Motor time (ms)	0.209	0.197	0.183	0.514	0.373	0.519	0.260	0.202	0.274
Speed of eye-hand reaction (ms)	0.204	0.168	0.198	0.308	0.204	0.365	0.198	0.100	0.247
**Peripheral Perception Test**									
Visual field(°)	−0.232	−0.189	−0.234	0.313	0.182	0.302	−0.156	−0.042	−0.233
Speed of eye-foot reaction (ms)	0.214	0.196	0.222	0.683 *	0.691 *	0.478	0.621*	0.544	0.634 *
Tracking deviation (pixels)	−0.088	−0.039	−0.156	0.032	0.021	−0.043	−0.119	−0.097	−0.181

*Note.* OSI-overall stability index. API-anterior-posterior index. MLI-medial-lateral index. * *p* < 0.05.
